# Piperacillin-Induced Immune Hemolytic Anemia in an Adult with Cystic Fibrosis

**DOI:** 10.1155/2010/161454

**Published:** 2010-06-02

**Authors:** Mahesh Bandara, David B. Seder, George Garratty, Regina M. Leger, Jonathan B. Zuckerman

**Affiliations:** ^1^Department of Medicine, University of Vermont and Maine Medical Center, 22 Bramhall St., Portland, ME 04102, USA; ^2^American Red Cross Blood Services, Southern California Region, Pomona, CA 91768, USA

## Abstract

We report a case of drug-induced immune hemolytic anemia (DIIHA) in an adult female with cystic fibrosis (CF), complicating routine treatment of a pulmonary exacerbation with intravenous piperacillin-tazobactam. Workup revealed a positive direct antiglobulin test (DAT) due to red blood cell (RBC)-bound IgG and C3 and piperacillin antibodies detectable in the patient's serum. The potential influence of CF transmembrane conductance regulator mutations on the severity of DIIHA is discussed. This report illustrates the importance of early identification of DIIHA, a rare complication of a commonly utilized medication in CF.

## 1. Introduction

Chronic infection with bacteria such as *Pseudomonas aeruginosa* in CF patients typically leads to progressive decline in lung function and early mortality [1]. Recent studies have indicated that more aggressive treatment of respiratory pathogens with intravenous antibiotics potentially results in better outcomes in affected individuals [2]. Piperacillin-tazobactam is an acylureidopenicillin beta-lactamase inhibitor combination with extended spectrum activity against *Pseudomonas aeruginosa *and is frequently used to treat pulmonary exacerbations. Piperacillin-tazobactam is an uncommon cause of drug-induced immune hemolytic anemia (DIIHA). We present a case of fulminant intravascular hemolysis due to piperacillin-tazobactam in a CF patient, review prior reports of this phenomenon, and discuss possible mechanisms underlying the clinical presentation of DIIHA in the CF population. Clinicians should recognize this life-threatening complication of routine therapy for CF pulmonary exacerbations and understand fundamental aspects of supportive care. This case has been previously reported in abstract form [3].

## 2. Case Report

### 2.1. Presentation

A 33-year-old, 52.9 kg woman with CF (genotype delta F508/ R560T) was admitted with increased chest congestion, exertional dyspnea, sputum production, fatigue, and low-grade fever, symptoms characteristic of a pulmonary exacerbation. Based on the antimicrobial susceptibility profile obtained on prior outpatient sputum cultures, she received piperacillin-tazobactam (4.5 gm IV every 6 hours), ciprofloxacin (400 mg IV every 12 hours), azithromycin (500 mg by mouth three times per week chronically as part of her outpatient regimen), and trimethoprimsulfamethoxazole (800 mg trimethoprim component by mouth twice daily) for the treatment of *Pseudomonas aeruginosa* and *Stenotrophomonas maltophilia*. She had previously received each of these medications without adverse effect during other hospitalizations. During the night of her seventh hospital day, she experienced nausea and violent vomiting. She complained of severe pain in the back, arms, and chest. She also described weakness and paresthesias in her extremities and a small blur in the left inferotemporal visual field. On examination, the patient was anxious, pale, and diaphoretic. There was no fever, and vital signs included heart rate of 110 beats/min, blood pressure of 106/65 mmHg, respiratory rate of 23 breaths/min, and oxyhemoglobin saturation of 100% while breathing room air. The precordium was hyperdynamic, but her skin was cool, with livedo reticularis over the thighs, knees, and calves. Over the subsequent two days, the urine became dark, with a pink hue.

### 2.2. Diagnostic Studies

The electrocardiogram and chest radiograph were unchanged. Laboratory evaluation disclosed a hemoglobin level of 4.8 g/dL, decreased from 12.2 g/dL two days earlier. Arterial blood analysis showed that the carboxyhemoglobin level was 5.3%. CT scan of the chest and abdomen did not reveal any acute process, and she was transferred to the intensive care unit for further management. Repeat blood count verified that the hemoglobin level was critically low at 4.3 g/dL, and the blood smear demonstrated stacked spherocytes. The lactate dehydrogenase (LDH) was 418 U/L, haptoglobin <20 mg/dL, and total bilirubin 2.4 mg/dL. The direct antiglobulin test (DAT) was strongly positive due to both IgG and C3d. The plasma-free hemoglobin level was elevated. The following day the reticulocyte count was 17.5% (index 2.36). Autoimmune hemolytic anemia (AIHA) was suspected, and no fully compatible unit of packed red cells could be identified by the blood bank. The vitamin E level was 4.9 mg/L (normal range 3.0–15.8 mg/L). Seven days after the reaction, a sample of her serum was sent to American Red Cross Blood Services in Pomona, California, where piperacillin antibody was detected by the “immune-complex” method, as previously described [4, 5]. Briefly, the patient's serum was tested with and without the presence of a 1 mg per mL solution of piperacillin against untreated and enzyme-treated RBCs. The patient's serum caused direct agglutination of e+ RBCs, but not of e− RBCs in the presence of the drug; the e− RBCs were, however, sensitized in the presence of the drug. Enzyme-treated RBCs, both e+ and e−, were slightly hemolyzed and strongly directly agglutinated in the presence of the drug. The negative controls were nonreactive. Thus the antipiperacillin demonstrated relative anti-e specificity. 

### 2.3. Clinical Course

Upon transfer to the intensive care unit, all antibiotics were discontinued, and the patient was treated with intravenous crystalloid, methylprednisolone, morphine, and transfusion of least-incompatible blood. Chest pain and nausea resolved within 24 hours. She received one dose of pooled immune-globulin (IVIG). Following these treatments, blood counts recovered back to baseline over three weeks, as illustrated inFigure 1. The carboxyhemoglobin level returned to normal, and repeat DAT performed thirteen days later was negative. Funduscopic examination by an ophthalmologist was described as normal, except for a possible cotton wool spot in the superior aspect of the left retina. The patient remained weak and had persistent joint pains, but her general condition improved with physical therapy, and the reported visual disturbance slowly improved over several weeks. She was discharged to home after 1 month.

## 3. Discussion

DIIHA may be a life-threatening complication of antibiotic therapy. Our patient's acute chest, back, and joint pain, accompanied by a rapid drop in hemoglobin and evidence of systemic hypoperfusion, reflects a fulminant, life-threatening hemolytic process. A Naranjo ADR Probability Score of 5 indicates probable association of piperacillin-tazobactam administration with the emergence of acute hemolysis in this case [6]. Review of existing case reports suggests that a severe clinical presentation may be characteristic of piperacillin-induced immune hemolytic anemia in patients with CF (see Table 1).

The antibiotics most frequently associated with DIIHA are cephalosporins (most commonly cefotetan and ceftriaxone), although over 100 different medications have been directly implicated [14, 15]. Reports of DIIHA attributed to penicillins in combination with *β*-lactamase inhibitors, such as piperacillin-tazobactam, ticarcillin-clavulanate, and ampicillin-sulbactam, are increasing [14, 16]. The literature review reveals only piperacillin and piperacillin-tazobactam as reported causes of DIIHA in patients with CF.

Table 1 shows clinical details of ten reported cases of piperacillin-induced immune hemolytic anemia. Four of these cases occurred in patients with CF, raising the question of whether piperacillin-related DIIHA has a different pathophysiology or higher incidence in the CF population, or perhaps simply reflects detection or reporting bias in an intensively monitored population.

DIIHA has occurred in patients after 7–13 days of therapy with piperacillin or piperacillin-tazobactam. The clinical syndrome is one of acute and severe anemia, which may be accompanied by hemoglobinuria, nausea, chest, back or joint pain, diaphoresis, headache, weakness, and paresthesias. Laboratory studies usually show anemia with spherocytes (predominantly extravascular hemolysis) or schistocytes (intravascular hemolysis). Carbon monoxide is a product of hemoglobin breakdown, created in equimolar amounts when bilirubin from the alpha methene bridge of heme is catabolized. Carboxy-hemoglobin levels are commonly elevated during hemolysis, providing an immediate clue to clinicians about the presence of DIIHA [17]. Serum tests available in most hospital laboratories typically indicate a decreased haptoglobin, increased free hemoglobin, increased LDH, total bilirubin and unconjugated bilirubin, and positive DAT for IgG and C3d. Urine will have an increased urobilinogen and free hemoglobin. Piperacillin antibodies may show a preference for Rh(e)+ red blood cells (RBCs) [3, 8, 18]. 

Continuation of the culprit medication may be lethal [4]. Patients benefit from rapid initiation of supportive care, including intravenous crystalloid to restore intravascular volume and blood transfusion to restore systemic circulation and oxygen carrying capacity. Although corticosteroids and/or IVIG are often given, there is no good evidence that they help suppress immune-mediated hemolysis when drug-dependent antibodies are involved; most reports of successful response to steroids involved simultaneous discontinuation of the drug, which was probably responsible for the resolution of hemolysis. Steroids may help when drug-induced autoantibodies are implicated; these cases are rarer than those caused by drug-dependent antibodies. We treated our patient with steroids and IVIG because the initial serology supported a diagnosis of AIHA. Later, the piperacillin antibodies were detected and no RBC autoantibodies were present. We believe that the initial results in which the patient's serum reacted with all e+ RBCs, without drug being present, were due to circulating drug-anti-drug complexes mimicking autoantibody. This phenomenon has been described previously [14, 16, 19]. Shirey et al. [10, 12] and Johnson [19] warned about confusing piperacillin-induced hemolytic anemia with a hemolytic transfusion reaction or AIHA, respectively.

The exact pathophysiology of DIIHA has yet to be fully elucidated. A number of possible mechanisms have been recently described and reviewed [14]. Drug antibodies may be directed at the drug or a combination of drug and erythrocyte membrane protein [14]. Such antibodies may combine with drug on the RBC membrane, either triggering extravascular hemolysis through interaction with macrophages in the reticuloendothelial system or initiating acute intravascular hemolysis via complement activation. While DIIHA is usually associated with extravascular hemolysis, intravascular hemolysis has been reported with many drugs (e.g., cefotetan and with ceftriaxone, especially in children) [14, 16]. All but one of the cases involving piperacillin in which serology or clinical presentation have been described implicate a complement-mediated intravascular process. Although our patient demonstrated clinical evidence of massive hemolysis, the LDH and total bilirubin were only modestly elevated, suggesting a predominantly extravascular process.

A mechanism that may influence the pathophysiology of hemolytic anemia in CF patients involves local nitric oxide (NO) production in the microcirculation. NO is a potent vasodilator, allowing RBCs to easily navigate microvascular interstices. Under normal circumstances, RBCs express ATP in response to mechanical stress on the cell membrane, which in turn upregulates endothelial cell NO synthesis [20, 21]. The cystic fibrosis transmembrane conductance regulator (CFTR), surface-expressed in RBCs and coupled to cytoskeletal elements, is essential to transcellular ATP movement [21]. Red cells from individuals with CF release markedly reduced levels of ATP in response to deformation leading to decreased endothelial NO production [21]. This abnormal signaling is compounded in the setting of hemolytic anemia, where free hemoglobin further scavenges NO in the microcirculation [22]. We hypothesize that due to these factors, DIIHA carries an enhanced risk for intravascular “sludging” and tissue ischemia in individuals with CF. This might explain the severe clinical course observed in the described patient, who has a genotype that results in significantly reduced functional CFTR expression [23].

While the details of membrane abnormalities in the erythrocytes of CF patients and their significance remain to be fully understood, it is imperative that clinicians recognize DIIHA and immediately discontinue potentially causative medications. We recommend formal immunohematologic characterization of DIIHA in CF patients. Confirmatory serological testing (testing of patient's serum in the presence of a solution of piperacillin against untreated and enzyme-treated RBCs) may be particularly useful in affected individuals [5]. It is important to clearly define DIIHA in order to safely guide future therapy in a patient population, where selection of appropriate antibiotics often becomes increasingly difficult over time.

## Figures and Tables

**Figure 1:**
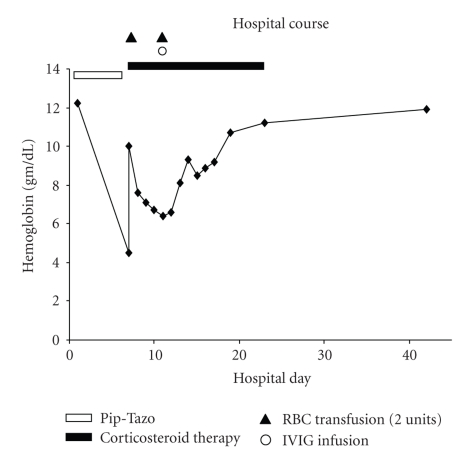
Hemoglobin profile over the hospital course. Timing of medical interventions is indicated by symbols (two units of packed red blood cells were given with each transfusion).

**Table 1:** 

Reference	Year	Age/Sex	Cystic fibrosis	Days to onset of symptoms	DAT	IgG	C3	Reported presentation	Treatment	Outcome
Thicket et al. [7]	1999	34/Female	Yes	13	+	NR	NR	AA, HU, N	Transfusion, steroids, folate, Pip-Tazo discontinued	Survived
Arndt et al. [4]	2002	29/Male	Yes	11	+	+	+	AA	Transfusion, Pip-Tazo discontinued	Survived
Arndt et al. [4]	2002	37/Male	Yes	12	+	Weak+	Weak+	AA, HU, RS	Transfusion, delayed Pip-Tazo discontinuation	Died
Seder and Zuckerman [3]	2006	33/Female	Yes	7	+	+	+	AA,HU, N	Transfusion, Pip-Tazo discontinued, steroids, immunoglobulin	Survived
Johnson et al. [8]	1994	46/Male	No	7	+	+	+	AA	NR	NR
Audeh and Wehrli [9]	2002	46/Female	No	7-9	+	+	−	AA	NR	Died
Shirey et al. [10]	2005	62/Female	No	NR	+	+	−	AA, HU, RS, N	Transfusion, Pip-Tazo discontinued	Survived
Mohammed and Greer [11]	2005	58/Male	No	2	+	+	+	AA	Transfusion, Pip-Tazo discontinued	Survived
Shirey et al. [12]	2007	68/female	No	1	+	+	Weak+	AA, HU	Transfusion, Pip-Tazo discontinued	Survived
Garcia Gala et al. [13]	2009	55/male	No	6	+	+	NR	AA	Transfusion, Pip-Tazo discontinued	Survived

AA = Acute anemia

HU = Hemoglobinuria

NR = Not reported

N = Nausea and/or vomiting

Pip-Tazo = Piperacillin-Tazobactam

RS = Respiratory symptoms.
